# Application Results of an Extracorporeal Therapy Protocol in Cardiorespiratory Arrest: A Historical Cohort Study

**DOI:** 10.3390/jcm14061842

**Published:** 2025-03-09

**Authors:** Jordi Castillo-Garcia, Albert Ariza-Solé, Eric Moral-González, Fabrizio Sbraga, Albert Gil-Dorado, Jose-Carlos Sánchez-Salado

**Affiliations:** L’Hospitalet de Llobregat, Carrer de la Feixa Llarga s/n, 08907 Barcelona, Spain; aariza@bellvitgehospital.cat (A.A.-S.); ericmoral@uic.es (E.M.-G.); sbraga@bellvitgehospital.cat (F.S.); agil@bellvitgehospital.cat (A.G.-D.); jcsanchez@bellvitgehospital.cat (J.-C.S.-S.)

**Keywords:** extracorporeal membrane oxygenation, cardiorespiratory arrest, cardiopulmonary resuscitation, survival, protocol

## Abstract

**Background/Objectives**: This study sought to evaluate the clinical profile, in-hospital management, prognosis, and survival of patients treated for cardiac arrest using extracorporeal therapy in a third-level Spanish hospital before and after the therapy was protocolised. **Methods**: This study is a historical single-centre cohort study that was conducted from January 2009 to February 2024. In 2019, an in-hospital extracorporeal reanimation therapy protocol was established in the centre’s Coronary Intensive Care Unit. As a result, the cohort was split into two groups: the Pre-Protocol group (between 2009 and December 2018) and the Post-Protocol group (between 2019 and February 2024). **Results**: A total of 26 patients were recruited, i.e., 10 in the first cohort and 16 in the second, with acute myocardial infarction being the most prevalent cause in both cohorts. A 30% (3) to 43.65% (7) increase in survival was observed between the two cohorts (*p* = 0.48), with CPC 1–2 neurological functionality exceeding 85% of cases in both cohorts (*p* = 0.7). The mean time from cardiac arrest to the application of extracorporeal therapy decreased from 104.1 min to 41.87 min (*p* = 0.09). The longer duration of ECMO (*p* = 0.03) and the longer hospital stay (*p* = 0.002) are due to a higher survival. **Conclusions:** The results show a trend in improvement outcomes. The small cohort size makes it difficult to draw robust conclusions, but we want to highlight the importance of applying a specific protocol based on standardised patient selection criteria and the establishment of extracorporeal reanimation therapy.

## 1. Introduction

It is estimated that the incidence of cardiac arrest (CA) in Spain is 1.5–2.8/1000 hospital admissions, approximately 24,000 cases per year [1,2].

Cardiovascular disease continues to be the main cause of CA aetiology, with acute ischaemic heart disease being the most frequent reason [3]. Despite scientific and educational advances in cardiopulmonary resuscitation (CPR) techniques, the implementation of CPR protocols and organisation in health centres has not significantly improved survival rates. Currently, the survival rate for in-hospital cardiac arrest (IHCA) remains between 15% and 34% at 30 days post-event [2,3,4], accompanied by heterogeneous neurological sequelae and the unknown but likely considerable consumption of health resources.

Refractory CA is defined as CA that persists after 10 min of advanced CPR [5,6]. It is a situation in which the survival rate is still quite low (5%) and continues to decline after 15 min [7], with the initial defibrillatable heart rate being associated with the best prognosis [3].

In order to improve the results of IHCA, several treatment strategies have been explored in the last decade. In 2015, these strategies were endorsed by the guidelines of the European Resuscitation Council (ERC) [8], where CA combined with the application of extracorporeal membrane oxygenation (ECMO) emerged as a way of boosting cardiac output to maintain the perfusion of vital organs and reduce hypoxia due to ischaemia [9], allowing for more time to identify and treat the cause of the CA [10].

In this highly complex scenario, the use of ECMO in CPR (ECPR) has shown improvements in the in-hospital survival rates of patients with refractory CA [11,12]. These survival rates can reach up to 50% when ECPR therapy is started within 30 min of IHCA, falling to 30% when ECPR therapy is started between 30 and 60 min after the event [13]. Neurological outcomes also improve when ECPR is carried out early [14].

In Spain, the use of this therapy in refractory IHCA is scarce [15] and is usually conducted in a non-protocolised way with a survival rate of 30% (3). In 2019, an ECPR protocol [16] was created at our centre by the Hospital Expert Group led by the Interdisciplinary CPR Commission. The execution of the protocol [16] (based on updated literature, team training, and role assignment) implies changes to the clinical management of patients, professionals, and clinical outcomes. In the UK, a project to improve the quality and outcomes of ECPR has been recently implemented, and during the next year, survival reached 69.2% [17].

Our study sought to evaluate the clinical profile, in-hospital management, prognosis, and survival of patients treated using ECPR therapy in a third-level Spanish hospital after the protocolisation of refractory CPR therapy.

## 2. Materials and Methods

### 2.1. Study Design

A historical single-centre cohort study was conducted from January 2009 to February 2024. In 2019, the in-hospital ECPR protocol [16] was established in the centre’s Coronary Intensive Care Unit (CICU), resulting in the cohort being split into two groups—the Pre-Protocol group (between 2009 and December 2018) and the Post-Protocol group (between 2019 and February 2024).

The protocol [16] was drafted jointly by the hospital’s ECMO and CPR Committee after authorisation from the centre’s management. It was based on a search of the scientific literature, and the procedure was adapted to fit in with the hospital’s organisation and functions. During the year, processes and skills were developed through clinical simulation, and the programme was explained to the clinical services involved. The protocol also establishes the necessary material for ERCP, the implant technique and management of the patient in CPR, and the organization of the team, specifically indicating the work of each of its members.

All patients with refractory CA in the first cohort were included. In the second cohort, after applying the protocol, patients were excluded if they met the following criteria: instructions for no resuscitation; a time without blood flow exceeding 10 min or no blood flow for 5 to 10 min; an ETCO2 reading below 10 mmHg in the first determination; contraindications of anticoagulation; a known terminal disease (with survival of less than one year); severe peripheral vascular disease; moderate–intense cognitive impairment; chronic renal failure requiring dialysis; or severe previous pulmonary disease or liver cirrhosis with portal hypertension [16].

### 2.2. Study Settings

Participating hospital information: the hospital in this study is one of the reference centres for ECMO, handling processes requiring high technology and complexity in the Barcelona Metropolitan Area, which serves a population of over two million.

### 2.3. Study Population

The population included patients admitted to the CICU between 2009 and 2024 who have had a CA episode and have received ECMO therapy.

### 2.4. Intervention and Outcomes

Variables: The variables included the patient’s initial heart rate, CA aetiology, previous serum lactate, time of low blood flow (low flow), survival at discharge, neurological status assessed on the 5-level CPC (Cerebral Performance Category), recovery of heart rate, complications stemming from ECPR, and time of hospital admission. Age, sex, medical history, and reason for admission were also analysed. The turning point variable between the cohorts was the implementation of the in-hospital ECPR protocol before or after 2019.

The potential influence of confounding factors, such as changes in technology or staff experience over the study period, has not been explored. These factors could have affected the results independently of the protocol.

### 2.5. Data Collection

Data collection was carried out from the patients’ medical records; this was completed by healthcare professionals. Patients who met the exclusion and inclusion criteria were identified, and a new anonymous database was generated through a thorough review of their medical records; the data needed to conduct the study were gathered.

### 2.6. Data Analysis

Quantitative variables are expressed as the mean and standard deviation (SD) for data with a normal distribution or as the median and inter-quartile range (IQR) for those without. Statistical comparisons between groups were conducted using Student’s *t*-test or Mann–Whitney’s U-test. Qualitative variables are presented in absolute frequency and %, and comparisons between the two groups were conducted using the χ^2^ test. The *p*-values are two-tailed and considered statistically significant if *p* < 0.05. The SPSS 29.0 statistical software was used.

Ethical considerations: this study was approved by the hospital’s Clinical Research Ethics Committee (CEIC) (PR257/19).

## 3. Results

The Pre-Protocol group recruited 10 patients, and the Post-Protocol group recruited 16 patients. The two groups were homogeneous in age, sex, and medical history (Table 1).

The aetiology of the CA also showed similar behaviour between the two groups, with no statistically significant differences observed. Acute myocardial infarction (AMI) was the most prevalent cause in both groups (Table 2).

Despite not obtaining data on initial heart rhythms in the first cohort, in the Post-Protocol group, defibrillable rhythms were detected in 18.75% of patients (3) with a 100% survival rate. The survival rate of those who presented with asystole as an initial rhythm was 0% (Table 3).

Although no statistically significant data were identified, an increase in survival was observed between the two cohorts from 3 (30%) to 7 (43.65%) with *p* = 0.48, including normal or moderately affected neurological functionality (CPC 1–2) exceeding 85% of cases in both cohorts (Table 3). The patients’ mortality was mainly due to post-anoxic encephalopathy (40%) and multi-organ failure (30%), with the rest being due to various other causes (30%).

The mean time from CA to the application of ECMO therapy was more than halved in the second cohort. The patients who survived the CA event had time values of 34.42 min or less. The patients who formed the second cohort, however, had a significant increase in days of therapy, especially survivors, with a mean of 10.14 (SD, 9.7) days (Table 3).

Bacteraemia occurred in 3 (30%) of patients in the first cohort as a complication stemming from ECMO therapy. In the second cohort, 4 (24%) of patients had complications, including bacteraemia in three cases and insertion point bleeding in one case.

## 4. Discussion

The results of this study show the need to implement the ECMO protocol even in a tertiary hospital with a cardiac transplant programme and extensive experience in mechanical support therapies, including ECMO. Taking into account the sample size and the short time of implementation of the protocol, we can consider that it is a pilot or exploratory study. Patient survival after the application of the protocol rose to 43.75%, which was higher than the 31% rate reported by the Extracorporeal Life Support Organization (ELSO) [18] and the latest International CPR guidelines [19]. Notably, the programme was applied in the Coronary ICU, which shows the cardiological patient profile—unlike the data published in an international series [18].

Although not statistically significant, the most striking data of our study relate to the clear reduction in the time taken to apply ECMO and the increase in patient survival.

It should also be noted that protocol implementation entailed excluding complex patients and the limitation of treatment in the second cohort. Another point to highlight is the strength of the protocol implemented [16]. This protocol was published to share key elements from the majority of the studies [20,21] aimed at achieving better results from ECPR therapy, such as the presence of CA and the early initiation of CPR manoeuvres. Like with other authors [1,18], this study also stresses CPR quality, the application of advanced life support manoeuvres, initial defibrillatable rhythm, the age of the patient, and the underlying aetiology of CA.

The sample of patients obtained presents mean ages and aetiologies of CAs that were similar to those reported in the published series [22]. The sample includes 26 patients, which is close to the 27 patients in Lin et al.’s study [23] but fewer than the 46 patients in Cheng et al.’s study [22]. It should be noted that our sample size is small, and the results should be seen as a pilot study for fostering the continuation of the programme or the development of other similar programmes.

The mean onset time for ECMO was 41.87 min, which is lower than the 43.8 min reported in Garcia et al.’s study [15], where the discharge survival rate of 25% of patients was slightly higher than the 26 min reported in Ohbe et al.’s study [20] but with a lower patient survival rate (20.5%) than that in our sample. In both of the cited studies, we saw no evidence that any type of protocol was implemented.

Our results provide average ECPR onset times of 34.42 min in surviving patients, which is very close to the 30 min recommended by most of the authors from the literature [11,13,20,21,24]. These times would not have been possible without the training of the protocol through clinical simulation [16], which is capable of reducing both the reaction and action time.

Significant results showing the protocol’s effectiveness include the increase in the days in ECMO therapy (10.14 days) and hospital stay for surviving patients (100.85 days), which will inevitably lead to greater consumption of both material and human resources. Such resource usage needs to be considered. Werner et al. study [25] estimated a range between USD 20,534 and 298,727 for each ECPR undertaken. The costs generated by these patients may grow when there are complications stemming from therapy, such as those seen in our patients. Complications such as bacteraemia or bleeding are similar to those mentioned by other authors [11,13,26,27].

The results show a 100% survival in patients with an initial defibrillatable rhythm. These results are shared by the international guidelines [19] and by other authors in the literature [28,29,30,31]. The initial defibrillatable rhythm must be a positive predictor when selecting patients in the protocol; meanwhile, they are a negative predictor if the rhythm is asystole (where we obtained a 0% survival rate). Few defibrillatable rhythms were found in the second cohort because they were treated quickly. Patients without major heart disease generally recover quickly, while those with underlying heart disease can rapidly progress to subsequent CA with pulseless electrical activity (PEA). Most patients present with PEA, indicating that their aetiology at admission is advanced heart disease in most cases; this means that their prognosis will be largely dependent on their type of heart disease and the treatment they receive for it rather than any neurological factors related to the CA. A percentage of 40% is a good survival rate in this scenario. This finding also justifies longer ECMO therapy and a longer stay in the CICU compared to patients with a defibrillatable rhythm. It should be noted that two of the patients ended up transplanted, and one ended up with mechanical support other than ECMO. The value of helping patients with asystole is often questionable, except in special circumstances such as pulmonary thromboembolism, drowning, and hypothermia.

The results obtained after applying the ECPR protocol also showed a slight improvement in the CPC neurological function. We obtained results superior to CPC 1–2 in 85% of patients in the two cohorts, similar to the 83.3% reported in Blumenstein et al.’s study [13]. This performance was much better than the series that did not employ the use of a specific protocol or selection criteria for the sample, which yielded a CPC 1–2 of 11% [12]. In-hospital CPR is associated with a very favourable neurological prognosis, as most cases are witnessed cardiac arrests with early, high-quality CPR. In cases of refractory cardiac arrest, ECPR can be rapidly performed without the need to mobilise the patient in most cases. This means that even in an older population with more pathology issues than the out-of-hospital population, the results are not only better but can also be as good as those shown in our series.

Although the use of this therapy is spreading, patient selection criteria do not present a clear consensus among scientific societies, although more specific and strict inclusion criteria, such as those in the second cohort [16], may be one of the keys to boosting survival rates.

Further research with larger sample sizes and better control for confounding factors will confirm that the protocol truly improves clinical outcomes.

## 5. Conclusions

Our results show an improvement (even not statistically significant) in survival, highlighting the importance of applying a specific protocol based on standardisation in patient selection criteria and the establishment of ECPR therapy.

### Study Limitations

This study has some limitations, such as the small sample size, its retrospective design, and its observational nature, which makes it very hard to exclude selection biases. In any case, the data reflect the trend in the results of the last 15 years of ECPR work conducted at the hospital. Some of the data from the first cohort could not be obtained, such as patient history of atrial fibrillation or smoking, which are known risk factors for ischaemic heart disease and CA. As it was a single-centre study, the extrapolation of the data to a series of different profiles and management is likely limited, so results should be interpreted with caution.

We recognise the lack of statistical significance for survival and neurological outcomes, as well as the possible implications of longer hospital stays and ECMO durations.

In the in-hospital ECPR of a cardiological patient, the factors that improve survival lead to longer hospital stays as well as higher requirements for ICU admissions, continuity of care, a mechanical support programme, and cardiac transplantation.

Notwithstanding these limitations, we believe that this study provides novel, relevant information on the implementation and initial results of an ECPR programme in real clinical practice. Improving the management and prognosis of these extremely complex conditions of patients could have very worthwhile clinical, economic, and social consequences.

## Figures and Tables

**Table 1:** Clinical and sociodemographic characteristics of the patients.

	Pre-Protocol Group	Post-Protocol Group	*p*-Value
	(*n* = 10)	(*n* = 16)	
Age (years)	47.6 (15.6)	53.68 (10.4)	0.24
Sex Male	8 (80%)	14 (87.5%)	0.6
HTA	3 (30%)	8 (50%)	0.31
DLP	4 (40%)	8 (50%)	0.61
DM	4 (40%)	3 (18.75%)	0.23
Anterior AMI	6 (60%)	9 (56.25%)	0.85
COPD	1 (10%)	1 (6.23%)	0.72
AF	-	3 (18.75%)	-
Active smoker	-	7 (43.75%)	-

Values expressed in mean (standard deviation) and frequency absolute (%). HTA: arterial hypertension; DLP: dyslipidaemia; DM: diabetes mellitus; AMI: acute myocardial infarction; COPD: chronic obstructive pulmonary disease; AF: atrial fibrillation.

**Table 2:** Aetiology of cardiac arrests.

	Pre-Protocol Group	Post-Protocol Group	*p*-Value
	*n* = 10	*n* = 16	
AMI	6 (60%)	11 (68.75%)	0.65
Decompensated cardiomyopathy	2 (20%)	2 (12.5%)	0.6
Post-cardiac transplant	1 (10%)	2 (12.5%)	0.85
Post-cardiotomy	1 (10%)	1 (6.25%)	0.88

Values expressed in absolute frequency (%). AMI: acute myocardial infarction.

**Table 3:** Survival outcomes and clinical variables between both cohorts.

	Pre-Protocol Group	Post-Protocol Group	
	(*n* = 10)	(*n* = 16)	
Initial Cardiac Arrest Rhythm			Survival
Desfibrilable	-	3 (18.75%)	100%
PEA	-	10 (62.5%)	40%
Asystole	-	3 (18.75%)	0%
			*p*-value
ECMO Start Serum Lactate	-	11.61 (3.95)	-
Discharge survival	3 (30%)	7 (43.75%)	0.48
CPC 1–2	3 (100%)	6 (85.7%)	0.7
Time CPR to ECMO Start (min)	104.1 (102.7)	41.87 (14.96)	0.09
Time CPR to ECMO Start in vivo (min)	-	34.42 (15.38)	-
ECMO Duration (Days)	3 (3.8%)	8.31 (7.62)	0.03
ECMO Duration in vivo (Days)	-	10.14 (9.70)	-
Hospital Stay (Days)	19.53 (23.6)	53.31 (24.04)	0.002
Hospital Stay in vivo (Days)	-	100.85 (68.30)	**-**
Complications arising from therapy	3 (30%)	4 (25%)	0.83

Values expressed in mean (SD) and absolute frequency (%). PEA: pulseless electrical activity; ECMO: extracorporeal membrane oxygenation; CPC: Cerebral Performance Scale Categories; CPR: cardiopulmonary resuscitation.

## Data Availability

The datasets are available from corresponding authors upon reasonable request.

## Abbreviations

The following abbreviations are used in this manuscript:

CA	Cardiorespiratory arrest
HA	Hospital admission
CPR	Cardiopulmonary resuscitation
ERC	European Resuscitation Council
ECMO	Extracorporeal membrane oxygenation
ECPR	ECMO with cardiopulmonary resuscitation
CICU	Coronary intensive care unit
CPC	Cerebral performance category
ELSO	Extracorporeal Life Support Organization
AMI	Acute myocardial infarction
PEA	Pulseless electrical activity
